# Cartwheel assembly

**DOI:** 10.1098/rstb.2013.0458

**Published:** 2014-09-05

**Authors:** Masafumi Hirono

**Affiliations:** Department of Biological Sciences, University of Tokyo, Tokyo, Japan

**Keywords:** hub, spoke, SAS-6/Bld12p, Cep135/Bld10p, SAS-5/Ana2/STIL

## Abstract

The cartwheel is a subcentriolar structure consisting of a central hub and nine radially arranged spokes, located at the proximal end of the centriole. It appears at the initial stage of the centriole assembly process as the first ninefold symmetrical structure. The cartwheel was first described more than 50 years ago, but it is only recently that its pivotal role in establishing the ninefold symmetry of the centriole was demonstrated. Significant progress has since been made in understanding its fine structure and assembly mechanism. Most importantly, the central part of the cartwheel, from which the ninefold symmetry originates, is shown to form by self-association of nine dimers of the protein SAS-6. This finding, together with emerging data on other components of the cartwheel, has opened new avenues in centrosome biology.

## 1. Introduction

**Figure 1.**
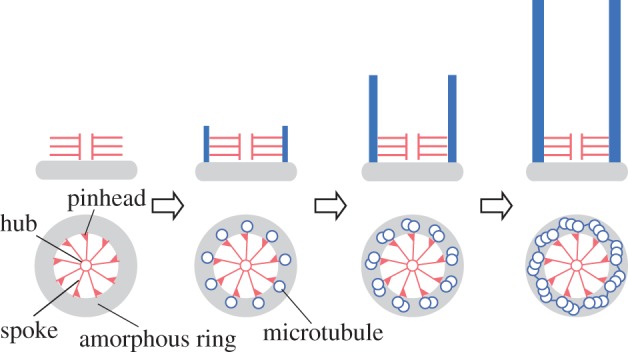
Cartwheel as the scaffold for centriole assembly. A cartwheel assembles on an amorphous ring or disc (grey) as the first ninefold symmetrical structure appearing in the centriole assembly process. Microtubules form at the tips of the nine spokes of the cartwheel. (Online version in colour.)

Centrioles and basal bodies (which will be referred to as centrioles unless distinction is necessary) have a characteristic structure with microtubules arranged in ninefold rotational symmetry. This structure has been faithfully reproduced over an infinite number of the organelle's duplication since the emergence of the last eukaryotic common ancestor and is strikingly well conserved among various present-day eukaryotic organisms. The precise structural conservation suggests that the nine-ness of the centriolar structure must be determined by a robust and conserved mechanism. At the centre of the mechanism is the cartwheel, a ninefold symmetrical structure that appears at the initial stage of the centriole assembly process (figure 1). It is more than 50 years ago that the cartwheel was first observed by electron microscopy [1], but only recently has great progress been made in understanding its function, molecular composition and assembly mechanism.

In this paper, I will summarize our current knowledge about the cartwheel structure and function. First, in §2, I explain structural features of cartwheels revealed by electron microscopy and cryo-electron tomography. In §3, I discuss the function of cartwheels in establishing the centriole's ninefold symmetry, and their possible other functions. Section 4 deals with molecular mechanisms of cartwheel assembly, focusing on two key proteins, SAS-6/Bld12p and Cep135/Bld10p. Section 5 explains what is known about the regulation of cartwheel and centriole assembly, by introducing the regulatory proteins identified to date. Finally, in §6, I discuss remaining important questions regarding cartwheel and centriole assembly. I have tried to introduce previous studies that are of particular interest but often neglected from recent literature, as well as the essential new findings made in this rapidly growing field.

## 2. Structure of the cartwheel

**Figure 2.**
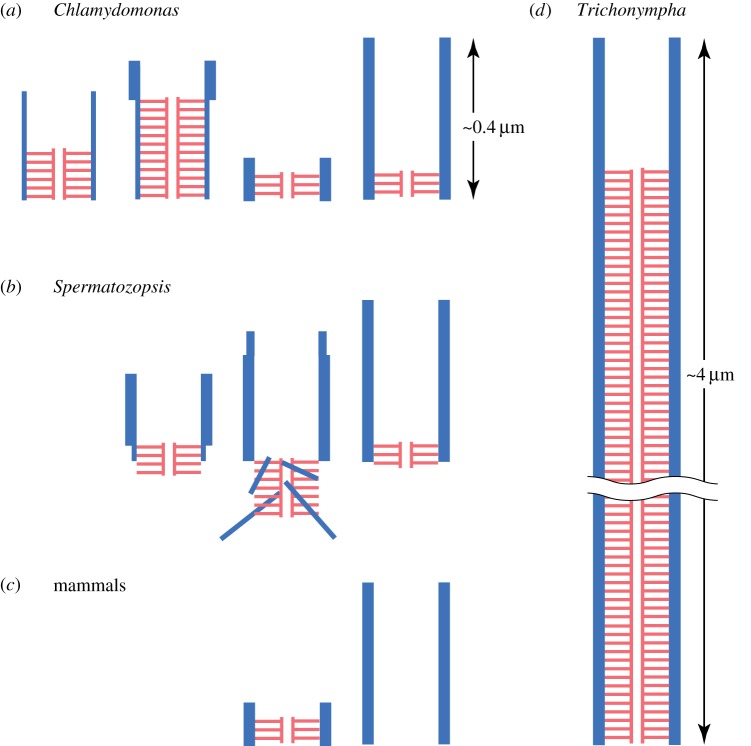
Divergence in length and position of cartwheel stacks (shown in red). (*a*) Dynamic change in the *Chlamydomonas* cartwheel stack during the centriole duplication cycle [18]. As the cell cycle progresses (from left to right), the lengths of the stack and microtubules change. (*b*) The cartwheel stack in *Spermatozopsis* also changes in length during the cell cycle. The cartwheel stack protrudes from the centriole lumen and apparently serves as a microtubule-organizing centre [16]. (*c*) In mammals, a cartwheel stack exists in the procentriole (left), but disappears from a fully assembled centriole (right). (*d*) The *Trichonympha* centriole is approximately 4 μm long, while the canonical centriole is approximately 0.4 μm long. The lumen except for the distal 10% is filled with cartwheels. Thin and thick blue lines indicate triplet and singlet microtubules, respectively. (Online version in colour.)

A single cartwheel is composed of a central ring (hub) from which nine filaments (spokes) emanate. Each spoke connects to the A-tubule of the triplet microtubule through a bulging structure called the pinhead. Usually, multiple cartwheels are stacked in the centriole lumen [1–15] (figure 1). The term ‘cartwheel’ is sometimes used to signify this entire stacked structure, but I will use the term for a single layer of this structure hereafter. While the basic structure and dimension of the hub and spoke are shared by most organisms, the number of the stacked cartwheels varies greatly among organisms and depending on the stage of centriole maturation. In most organisms, centrioles are 400–450 nm long and have several layers of stacks in the proximal approximately 100 nm of the organelle (figures 1 and 2*a*). However, an exceptionally long (approx. 4 μm) centriole is present in *Trichonympha* [1] and is shown to contain hundreds of cartwheel layers that fill approximately 90% of the lumen (figure 2*d*). A stage-dependent variation is found in *Chlamydomonas* and *Spermatozopsis* centrioles; centrioles in developing stages contain 7–10 layers of cartwheels, while those in the mature stage contain two to four layers (figure 2*a,b*) [16–18]. In mammalian centrosomes, cartwheels are present in the procentriole but disappear during mitosis (figure 2*c*) [8,19].

**Figure 3.**
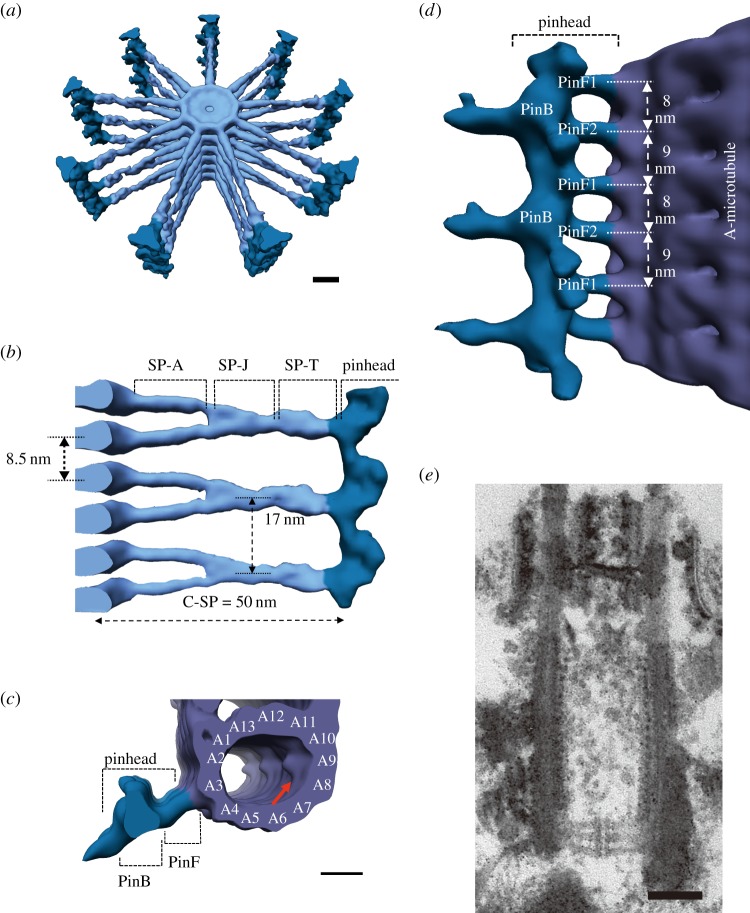
Cryo-electron tomography images of the cartwheel in *Trichonympha*. (*a*) Three-dimensional representation of a stack of cartwheels (light blue) with pinheads (dark blue). Each cartwheel is composed of nine approximately 50 nm long spokes emanating from the central hub (diameter: approx. 22 nm). Scale bar, 10 nm. (*b*) Side view of the cartwheel stack highlighting the cartwheel spoke (C-SP). Hubs (left-hand side) are stacked with an 8.5 nm periodicity. Spokes in the adjacent cartwheels are paired and merged at approximately 20 nm from the hub. The spoke is structurally divided into a spoke arm (SP-A), a spoke junction (SP-J) and a spoke tip (SP-T). (*c*) Top view of the pinhead and the A-tubule of the triplet microtubule (violet). The pinhead is subdivided into a pinbody (PinB) and pinfeet (PinF) that connect the pinbody to the microtubule. Scale bar, 10 nm. (*d*) Side view of the pinhead associated with the microtubule. The pinfeet consist of pinfoot 1 (PinF1) and pinfoot 2 (PinF2), which alternate every 8 and 9 nm along the microtubule axis. The tilting of the pinfeet towards the proximal end of the centriole defines the polarity of the cartwheel. Reproduced with permission from [21]. Copyright 2013 Elsevier. (*e*) A thin section image of the *Chlamydomonas* centriole. The cartwheel stack displays approximately 20 nm periodicity, a distance close to that of merged spokes observed in the *Trichonympha* cartwheel. Scale bar, 100 nm. (Online version in colour.)

Cryo-electron tomography determined the cartwheel structure of *Trichonympha* at a resolution of 38–42 Å (figure 3) [20,21]. This high resolution was achieved by averaging the images of a large number of stacked cartwheels. The reconstituted three-dimensional structure shows cartwheels occurring as stacked layers as previously thought, although there was a proposal that the extensive cartwheel stack might be produced by helical assembly of the components [22]. The central hub is a ring with a diameter of approximately 22 nm stacked with an 8.5 nm periodicity (figure 3*a*,*b*). The spoke is approximately 50 nm long between the ring and the pinhead. Interestingly, spokes in adjacent layers of cartwheel merge at approximately 20 nm from the ring, exhibiting a vertical periodicity of 17 nm, which corresponds to the size of two tubulin dimers in the microtubule protofilament (figure 3*b*,*d*). This periodicity is close to the approximately 20 nm periodicity of the cartwheel stacks observed by conventional electron microscopy in various organisms (figure 3*e*) [4,10,16,17]. Thus, merging of adjacent spokes, as observed in *Trichonympha*, may be a general feature of the cartwheel.

Another important finding from the tomography study is that the pinhead has a complex structure polarized in the direction of the centriole axis [21]. The pinhead consists of a hook-like protrusion (pinbody) and two linkers (pinfeet) present between the pinbody and the microtubule (figure 3*c*,*d*). The polarity is defined by a tilt of pinfeet towards the proximal end of the centriole (figure 3*d*). The presence of polarity is interesting because the central part of the cartwheel is composed of nine SAS-6 dimers (see §4*a*) and the dimer has a twofold symmetry with respect to the axis of the coiled-coil spoke; in other words, the core structure of the cartwheel has no polarity in the direction of the centriole axis [23,24].

**Figure 4.**
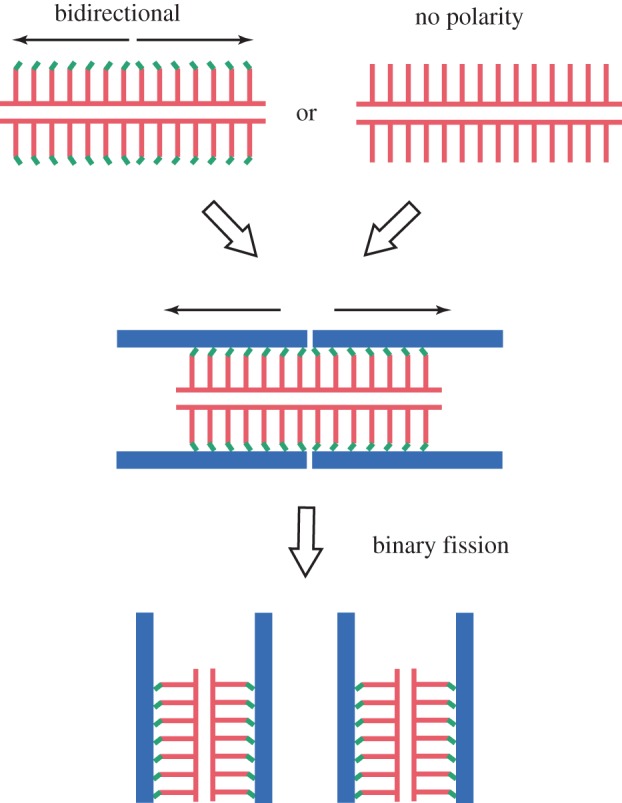
Characteristic centriole assembly process in bryophytes and a marine protist. Two nascent centrioles (blue lines) assemble on a cartwheel stack (red lines), with their proximal ends facing each other, and then separate through binary fission [13,14]. Two models can be thought of for the assembly of the cartwheel stack, which serves as the scaffold for the bidirectional assembly of two centrioles. One model postulates that cartwheels having pinheads (green ends of lines), each with structural polarity, assemble bidirectionally from a putative nucleator. The other model postulates that cartwheels first assemble without pinheads or polarity, and acquire pinheads and microtubules afterwards. (Online version in colour.)

Regarding the structural polarity of the cartwheel stack, interesting observations have been made in some bryophyte species [13] and a marine protist, *Labyrinthula* [14], which may provide an insight into the process of microtubule assembly on the cartwheel. The process of de novo centriole formation, which occurs during mitosis in these organisms, suggests that two new centrioles assemble bidirectionally from a preassembled cartwheel stack, and the resulting centrioles share the stack at their proximal ends (figure 4). Although it is possible that these centrioles (a bicentriole) are formed by joining of two mature centrioles through their proximal ends, like the colinearly arranged mother and daughter centrioles observed in quiescent mammalian cells [25], morphological markers of mitotic stages strongly suggests that, in these organisms, the bicentriole forms first and thereafter its binary fission produces two mature centrioles. Given that the cartwheel polarity resides only in the pinhead, and that the pinhead is composed of a component(s) different from the rest of the cartwheel as I will discuss later, it seems possible that the cartwheel stack may first assemble without pinheads and polarity, and pinheads and microtubules assemble on the cartwheel afterwards.

The cartwheel observed by conventional electron microscopy in an aquatic fungus, *Phlyctochytrium*, shows a thin filament at approximately 20 nm from the hub, the position where the two spokes in adjacent layers merge in the *Trichonympha* cartwheel [5]. This filament possibly functions as a cross-link between the spokes. In the *Chlamydomonas* cartwheel, similar but more irregular filaments are observed near the pinhead [26,27]. It is tempting to speculate that these filaments function to stabilize the radial arrangement of the nine spokes. In fact, the radial arrangement of spokes appears to be stable even without surrounding microtubules [6,28]. Whether these putative linking filaments are generally present in cartwheels of all species awaits further studies.

## 3. Functions of the cartwheel

### (a) A brief history

Since first described in 1960, the cartwheel has been found in the flagellar/ciliary basal bodies and centrioles of various organisms, including protists, algae, fungi and mammalian cells [1–15,29]. Extensive morphological studies on the centriole formation process revealed that, in certain species, the cartwheel appears before the appearance of nine singlet microtubules [6,9,14,30]. Together with its characteristic structure, this finding has led to the idea that the cartwheel determines the microtubule number of the centriole [30,31]. However, because the cartwheel was often regarded as an ‘only a morphologically described structure’ until recently, its function had long been controversial. Several cartwheel-independent mechanisms have been proposed to explain the assembly of the ninefold structure on the assumption that subcentriolar structures or precursor structures are responsible. For example, some studies proposed that the ninefold symmetry originates from the spacers that transiently appear between the microtubules [4], from the close packing of the triplet microtubules [32], or from the amorphous discs or rings that appear prior to the cartwheel [33]. Reasons for disregarding the cartwheel include: (i) the cartwheel appears after the formation of circularly arranged nine microtubules during basal body development in *Paramecium* [4]; (ii) no cartwheels have been observed in the centrioles of some organisms [32]; and (iii) in *Caenorhabditis elegans*, the central tube, a cylindrical structure that has no apparent ninefold symmetrical features, appears to function as the scaffold for the assembly of nine microtubules [33,34]. Although the above-mentioned alternative ideas may still help understand the mechanism of centriole's ninefold symmetry in some aspects as I will discuss later, these ideas have the obvious limitation that they are solely based on ultrastructural observation and not on molecular information.

### (b) Function in establishing ninefold symmetry

**Figure 5.**
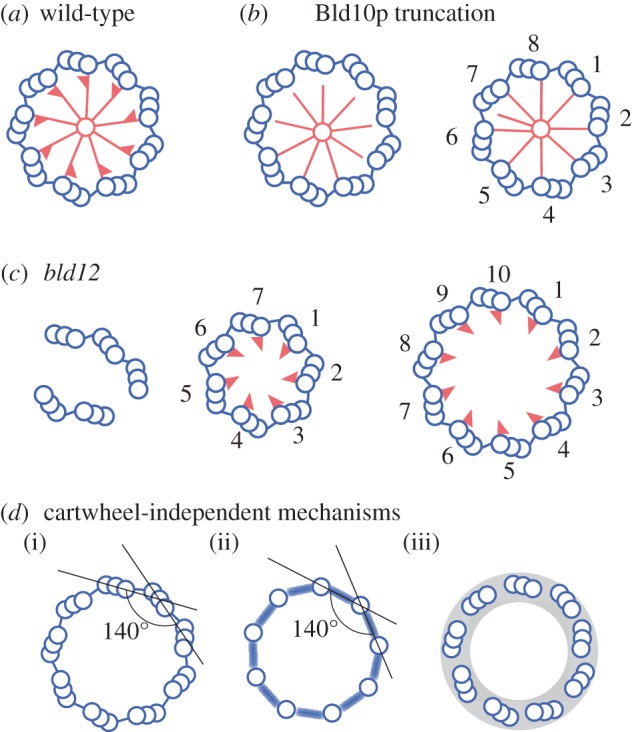
Cartwheel-dependent and -independent mechanisms for establishing the ninefold symmetry in the centriole. (*a*) Cartwheel and triplet microtubules arranged in ninefold symmetry in the normal centriole. (*b*) Abnormal cartwheels and microtubule arrangement in centrioles comprising truncated Bld10p. In *bld10* cells expressing a Bld10p without the N-terminal 54% or the C-terminal 35%, various deficiencies are observed. For example, centrioles frequently have only eight triplets, the spoke and its tip are shorter and thinner, and the spoke tips are often detached from the triplets [26]. (*c*) Abnormal centrioles formed in the absence of cartwheels in *bld12* cells. The majority of the centrioles are split in pieces of one to five triplet microtubules. The rest of the centrioles retain the circular arrangement, but have a variable number of triplets, ranging from seven to 11 [27]. (*d*) Speculations about cartwheel-independent mechanisms that produce ninefold symmetry. (i) If the linker connecting the A- and C-tubule of the triplets specifies the angle between the two triplet blades, nine microtubules would be on the centriole circle. (ii) Similarly, the linker connecting the singlet microtubules, which appears at an early stage of centriole assembly process [16], could specify the angle. (iii) An amorphous ring that appears before the formation of the cartwheel and restrict the loci for microtubule formation, may roughly determine the triplet number. (Online version in colour.)

The first experimental evidence for the importance of cartwheel function came from analyses of a *Chlamydomonas* mutant that totally lacks centriolar microtubules [26]. This mutant, *bld10*, has a null mutation in the gene coding for Bld10p, a coiled-coil protein homologous to a mammalian centrosomal protein, Cep135 [35]. Bld10p is localized to a region of the cartwheel spoke close to the triplet microtubule. Interestingly, when a truncated Bld10p is expressed in *bld10*, cartwheels with shorter spokes are produced and, furthermore, centrioles in these cells frequently have eight triplet microtubules rather than nine (figure 5*b*). The eight triplet centrioles are assembled most probably because the smaller circumference of the cartwheel defined by the shorter spokes can accommodate only eight triplets. These findings indicate that Bld10p is a component of the cartwheel spoke, and that the cartwheel structure plays an essential role in establishing the ninefold symmetry of the centriole. In the eight triplet centrioles, the cartwheel still retains nine spokes but one spoke is disconnected from a triplet microtubule (figure 5*b*). This indicates that the ninefold symmetry of the cartwheel originates from its central part including the hub [26].

The importance of the cartwheel was further supported by analyses of another *Chlamydomonas* mutant, *bld12* [27]. This mutant carries a null mutation in the gene coding for a homologue of SAS-6. (To avoid confusion with *bld10*, I will refer to this mutant as *bld12*(ΔSAS-6) in this review.) SAS-6 is a protein that was shown to be essential for centriole assembly in *C. elegans* and humans [19,36,37], and localized to the cartwheel in some organisms [37,38]. Electron microscopy revealed that the *bld12*(ΔSAS-6) mutant cells lack the central part of the cartwheel (figure 5*c*) and immunoelectron microscopy localized SAS-6/Bld12p to the same central part in the wild-type cartwheel. These findings indicate that this protein constitutes the hub and the central part of the spoke [27]. The majority of the *bld12*(ΔSAS-6) centrioles are split in pieces of one to five triplet microtubules, and fewer than 20% of all centrioles retain the circular arrangement of the triplets (figure 5*c*). Strikingly, these circular centrioles have variable numbers of triplets from seven to 11 (see below), suggesting that the central part of the cartwheel stabilizes the triplet number of the centriole (figure 5*c*).

Similar results are obtained in other organisms. In a *Drosophila* null mutant of the gene for SAS-6/Bld12p, and a *Paramecium* strain whose four genes for SAS-6/Bld12p have been silenced by RNAi, centrioles are formed with variable numbers of triplets. In both cases, the SAS-6/Bld12p protein is localized to the central region of cartwheels [39,40]. Likewise, in *Tetrahymena*, depletion of the *SAS6a* gene, one of the two SAS-6 genes present in this organism, causes defective centriole assembly owing to a loss of the cartwheel [41]. Together with the fact that SAS-6/Bld12p and Bld10p/Cep135 are conserved among organisms that have centrioles [42], these results strongly suggest that the cartwheel generally functions as a scaffold for centriole assembly and stabilizes ninefold symmetry. The observation that some organisms lack cartwheels in their centrioles might seem to argue against the general importance of the cartwheel [32]. However, it is likely that the lack of cartwheels in these organisms is owing to their disassembly during maturation of the centriole [8].

The phenotype of *bld12*(ΔSAS-6) is indicative of an important feature regarding how the ninefold symmetry is produced. That is, approximately 70% of the circular centrioles assembled in *bld12*(ΔSAS-6) are still composed of nine triplets even though the central part of the cartwheel is completely missing [27]. This suggests that an unknown mechanism(s) not involving the cartwheel also functions in the establishment of the centriole's ninefold symmetry. I speculate that the putative cartwheel-independent mechanism roughly determines the triplet number around nine, and the cartwheel-dependent mechanism fixes the number to nine. A possible structural component for the cartwheel-independent mechanism could be the linker that connects between the A- and C-tubules of the two adjacent triplets [4], or between the singlet microtubules that appear at an early step of centriole assembly [16] (figure 5*d*). If these linkers specify the interior angle of the polygon formed by triplets or singlets to 140°, nine microtubules would be on the centriole circle (figure 5*d*) [43]. In addition to the A–C linker, an amorphous ring or disc that appears before the formation of the cartwheel during centriole development may also contribute to establishment of the ninefold symmetry [33]. If the triplet assembly sites are restricted to the circumference of the amorphous ring, the size of the ring would roughly determine the triplet number (figure 5*d*). For an understanding of the complete mechanism that establishes the ninefold symmetry, we must explore both the cartwheel-dependent and -independent mechanisms.

### (c) Other possible functions

In addition to the function in establishing ninefold symmetry in the centriole, the cartwheel appears to maintain or strengthen the cylindrical arrangement of the triplet microtubules. Knockout of the *CEP135/BLD10* gene in *Tetrahymena* causes disassembly of pre-existing centrioles, as well as inhibition of new centriole assembly. Interestingly, disassembly does not occur if vigorous ciliary motility is inhibited, suggesting that the cartwheel contributes to the structural strength of the centriole [44]. In *Drosophila* also, the cartwheel appears to hold the triplets tight, because the diameter of the centriole is wider in mutants lacking Cep135/Bld10p (this protein is not essential for centriole assembly in this organism, see §4*b*) [45]. Together with the observation that the majority of the *bld12*(ΔSAS-6) centrioles are fragmented, these results suggest a cartwheel's function as an inner skeleton of the centriole. Furthermore, it should be noted that such deteriorating effects of cartwheel loss are not observed in mammalian centrioles, which naturally lose the cartwheel as cell cycle progresses. Mammalian centrioles may be stabilized by mammalian-specific centriole components instead of the cartwheel.

The phenotype of the above-mentioned *Drosophila* mutant implies another possible function of the cartwheel. In wild-type *Drosophila*, one of the central pair of microtubules in sperm flagella nucleates from the centriole lumen. However in this mutant, the single microtubule in the centriole and the central pair of microtubules in the flagellar axoneme do not form [46]. Although direct association has not been observed between the cartwheel and the microtubule end, their proximity suggests that the cartwheel may function as a nucleator of one of the central pair of microtubules [46]. However, the nucleating function of the cartwheel, if any, may be limited to some species, because penetration of the central microtubule into the centriole is known only in *Drosophila* [47–50].

## 4. Molecular mechanism of cartwheel assembly

### (a) Cartwheel central part assembled from SAS-6/Bld12p

**Figure 6.**
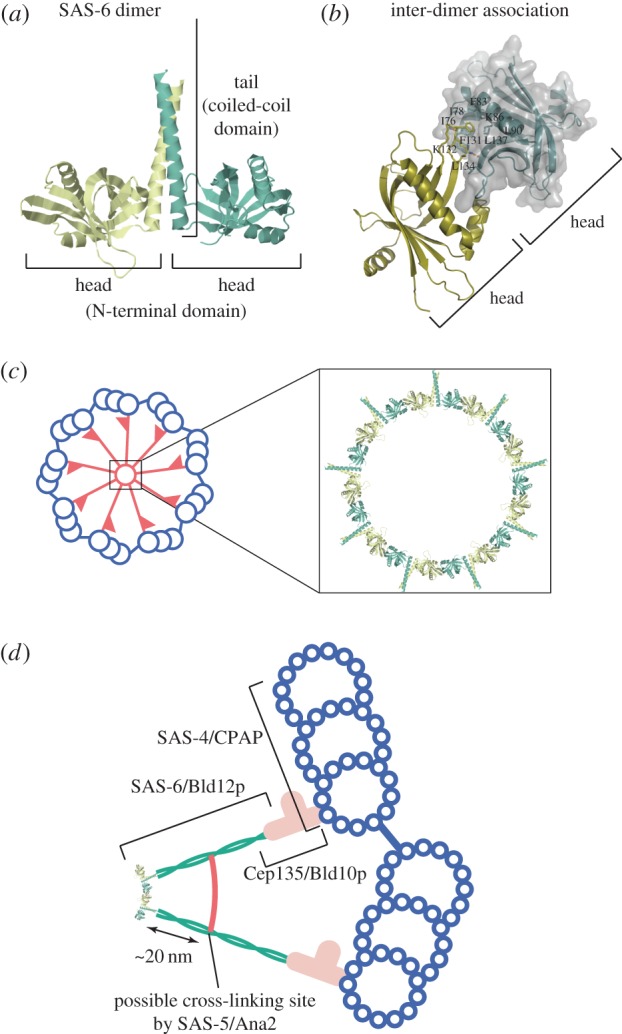
The three-dimensional structure of SAS-6 dimer and a model of SAS-6 assembly into the cartwheel [23,24]. (*a*) The structure of a zebrafish SAS-6 fragment that contains the N-terminal domain and part of the coiled-coil domain. The fragment forms a homodimer consisting of two globular heads and a coiled-coil tail. (*b*) The site of inter-dimer association in zebrafish SAS-6. The interaction is mostly between hydrophobic residues in the head domain. The hydrophobic Phe131 residue of one head is inserted into a hydrophobic pocket in the other head. Some of the residues that form the hydrophobic pocket are indicated. (*c*) SAS-6 ring model predicted by the structure. Nine SAS-6 dimers assemble into a ring with nine radiating filaments. This structure constitutes the central part of the cartwheel. (*d*) Localization of the cartwheel components. SAS-6/Bld12p constitutes the cartwheel hub and the central part of the spoke. Cep135/Bld10p constitutes the pinhead and a distal part of the spoke shaft and connects the cartwheel to the microtubules. Localizations of these proteins partially overlap. SAS-5/Ana2 may cross-link two cartwheel spokes at a site approximately 20 nm apart from the hub ring (see text). SAS-4/CPAP is localized around the microtubule. Localization of STIL, which binds to CPAP, is not established. (Online version in colour.)

As discussed above, several lines of evidence indicate that SAS-6/Bld12p is a component of the central part of the cartwheel. What mechanism assembles this protein into the ninefold symmetrical structure? The answer came from X-ray crystallography and biochemical analyses performed independently by two groups [23,24]. The two groups used SAS-6/Bld12p of three different organisms, *C. elegans*, *Chlamydomonas* and zebrafish, but the results obtained are similar to each other, indicating the conserved function of this protein [43]. SAS-6/Bld12p consists of a conserved N-terminal domain, a coiled-coil domain and a non-conserved C-terminal domain. Crystallography and biochemical analyses of a fragment containing the N-terminal domain attached with part of the coiled-coil domain, and another fragment containing the N-terminal domain alone, revealed that this protein forms a stable homodimer via association of the coiled-coil domains to form a long coiled-coil tail (figure 6*a*), and that the dimers interact with each other through a hydrophobic interaction between the head domains (figure 6*b*). In the latter interaction, an Ile (*C. elegans*) or a Phe (*Chlamydomonas* and zebrafish) residue within a loop in one head is inserted into a hydrophobic pocket in the other head. Although this hydrophobic interaction is weak (*K*_d_ for *C. elegans*, zebrafish and human SAS-6s are approx. 110, approx. 90 and approx. 50 μM, respectively), mutational analyses demonstrated that it is essential for centriole assembly. These results led to a model in which nine dimers assemble into a ring through the head-to-head interaction, and the head ring and the coiled-coil tails constitute the hub and the spokes of the native cartwheel (figure 6*c*). In fact, SAS-6 dimers in solution form oligomers, including nonamers [24], and a ring structure having a diameter close to that of the cartwheel hub [23]. The model predicts that the C-terminus of the SAS-6 molecule should be localized distal to the hub. Immunoelectron microscopy of *Chlamydomonas* cells expressing Bld12p tagged with HA at the C-terminus confirms the predicted orientation [24,51]. Considering the cartwheel function in centriole assembly, all these findings collectively indicate that the oligomerization properties of SAS-6/Bld12p are crucial for stabilization of the ninefold symmetry of the centriole.

Centriole assembly in *C. elegans* does not conform to the cartwheel-centred pathway; as mentioned before, microtubules assemble around the central tube, instead of the cartwheel. The tube is composed of SAS-6 (ceSAS-6), which shares a common domain structure with other SAS-6 family members. Because the tube looks very different from the cartwheel—it is rather an amorphous tube with a diameter about half as large as that of the cartwheel—it is puzzling how it functions in the establishment of the ninefold symmetry of the centriole. However, a recent study proposed a model that can explain the mechanism [52]. In contrast to zebrafish SAS-6 or *Chlamydomonas* Bld12p forming dimers that self-assemble into a ring, X-ray crystallography of ceSAS-6 predicts that it forms dimers that will assemble into a right-handed helical filament with spokes projecting outwards. In fact, a ceSAS-6 variant, which is designed to display stronger inter-dimer association, assembles *in vitro* into a helical filament with approximately 31 nm pitch as predicted by the model, as well as a double-stranded filament with two tightly inter-wound helical filaments. Because the single-stranded helix has nine dimers every two turns, the single- or double-stranded helices display the ninefold radial symmetry every 62 or 31 nm. Thus, the single- or double-stranded helical filament could serve as the scaffold for the assembly of nine microtubules. In addition, the diameter and length of the central tube in the *C. elegans* centriole closely correspond to four turns of the single helix. This model provides an interesting example of potential deviation from the conserved mechanism based on the oligomerization of SAS-6.

### (b) Cartwheel distal part assembled from Cep135/Bld10p

For the establishment of the ninefold symmetrical centriole structure, the length of the cartwheel spoke is as important as the assembly of the ninefold symmetrical hub [53,54]. Experiments using *Chlamydomonas bld10* mutants expressing truncated Bld10p revealed that Bld10p constitutes the distal part of the spoke and contributes to the formation of spokes of the proper length (figure 5*b*) [26]. Bld10p is a 170 kDa protein that forms a dimer through parallel association of the coiled-coil domains that span almost its entire length (M. Hiraki 2008, unpublished data) [42,55]. A single coiled-coil dimer of this size, if fully extended, could span up to 145 nm, but Bld10p is localized to only approximately 40 nm of the distal part of the spoke. How is this protein folded at the spoke end? Immunoelectron microscopy of centrioles in *Chlamydomonas* and *Paramecium* shows that the N-terminus of Bld10p localizes near the triplet microtubule [26,40]. The spoke tips are often disconnected from the microtubules if the N-terminal 54% of Bld10p is truncated [26]. Thus, the N-terminal region appears to be where the microtubule attaches. In fact, N-terminal fragments of *Drosophila* Bld10p (residues 1–163) and human Cep135 (residues 1–190), which contain a conserved sequence in this protein family, have been shown to directly bind to microtubules [46,56]. These results suggest that the N-terminal portion of Bld10p constitutes the very tip of the spoke, probably forms the linker (pinfeet), and directly binds to the microtubule [21]. However, it should be noted that, in *Chlamydomonas*, the N-terminal 44% of Bld10p (residues 1–727) is almost fully dispensable for centriole assembly [26]. This observation suggests that a factor(s) other than the Bld10p N-terminus is involved in the spoke-microtubule connection in the centriole. In this regard, the two distinct pinfeet observed in a single pinhead [21] may well each represent the Bld10p N-terminus and an unidentified microtubule-binding factor. SAS-4/CPAP, a core centriole protein that binds with both microtubules and Cep135 [56–58], is a candidate for the factor [21].

Deletion of the C-terminal 35% of Bld10p (residues 1070–1640) reduces the spoke length and pinhead size, indicating that this portion constitutes part of the spoke shaft and the pinhead. The finding that Bld10p contributes to the spoke length suggests its direct interaction with the coiled-coil domain of SAS-6, which constitutes the spoke in the central part of the cartwheel. This idea is supported by immunoelectron microscopy observation that the C-terminal portion of Bld10p and the C-terminal portion of SAS-6 overlap [24,26]. Furthermore, a human Cep135 fragment (spanning the C-terminal 64%; residues 416–1140), expressed in cultured cells, directly binds to an SAS-6 fragment that includes the C-terminal portion of its coiled-coil domain and the non-conserved domain [56]. Hence it is conceivable that Bld10p extends the spoke by a direct association with the tail of the SAS-6 dimer, or possibly with an associated pair of tails [20,21], and helps connect the spoke to the microtubule (figure 6*d*). More details of the Bld10p folding at the spoke tip will certainly be clarified by cryo-electron tomographic analysis of cartwheels containing intact and truncated Bld10p.

Bld10p may function not only in tethering microtubules to the cartwheel but also in the assembly of centriolar microtubules. This is because the *Chlamydomonas bld10* mutant totally lacks centriolar microtubules [55]. Bld10p may recruit γ-tubulin ring complex, the complex that nucleates tubulin assembly, around the cartwheel [59–63]. The central part of the Bld10p sequence may be involved in this function because this part is crucial for the centriole formation and because significant portions of the N- and C-terminal sequences are dispensable for the Bld10p function [26].

Cep135/Bld10p is essential for centriole formation in humans, *Paramecium*, *Tetrahymena* [37,40,44,56]. However, this is not the case in some organisms. The Cep135/Bld10p homologue in *Drosophila* is localized all along the centriole microtubules, not restricted to the proximal end. Importantly, *Drosophila* mutants that lack the Cep135/Bld10p homologue assemble cartwheels and centrioles, although the cartwheel appears to be unstable [42,45,64,65]. Instead, they have short centrioles and lack central pair microtubules in sperm flagella. These observations, together with microtubule-binding activity detected in *Drosophila* and human Cep135/Bld10p [46,56], suggest that this protein functions in nucleating and/or stabilizing microtubules as a microtubule-associated protein rather than constituting part of the cartwheel spoke [46]. In DT40 chicken lymphoma cells, disruption of the Cep135 gene has almost no effects on cell proliferation other than a slight increase in appearance of monopolar spindle [66]. Although the dispensability of Cep135/Bld10p for centriole formation in these organisms must be studied further, it seems possible that *Drosophila* has a second Cep135/Bld10p-like protein. The microtubule-binding activity of Cep135/Bld10p may function in stabilizing flagellar microtubules in some organisms or cells other than *Drosophila* [46].

### (c) Other possible components of the cartwheel

Several proteins other than SAS-6/Bld12p and Cep135/Bld10p are localized to the cartwheel [34,38,67,68]. Of these proteins, SAS-5/Ana2/STIL has been shown to function in cartwheel assembly [69]. SAS-5 is an SAS-6-binding protein essential for centriole assembly in *C. elegans* [36,70,71]. Ana2 is a *Drosophila* protein whose overexpression induces over-duplication of centrioles [72]. STIL is a human protein whose mutation causes primary microcephaly (MCPH), a genetic disease to which several centriole proteins are linked [73–75]. Ana2 and STIL are thought to be functional orthologues of *C. elegans* SAS-5 despite poor sequence homologies and differences in molecular weight [68]. They are regarded as homologues because, in addition to all being essential for centriole assembly, all share two short sequences called STAN (*ST*il/*AN*a2) and TIM (*T*runcated *I*n *M*icrocephaly) motifs at the C-terminal regions [68,74,75]. Similar to SAS-5, Ana2 binds to SAS-6, suggesting its function in cartwheel assembly [68,73]. Strikingly, when overexpressed in *Drosophila* culture cells, Ana2 and SAS-6 coassemble into a cluster of cartwheel-like structures, which contain tubes similar to stacked hubs, and filaments radiating from the tubes [72]. STIL is also associated with SAS-6 in the centriole, although indirectly (through binding to CPAP). The STIL-CPAP binding is essential for centriole assembly; mutations that attenuate the association can cause MCPH [73,76–79]. Involvement of STIL in cartwheel assembly is further implicated by the observation that this protein is localized to the proximal end of the daughter centriole at the onset of centriole duplication [75]. These findings suggest that SAS-5/Ana2/STIL cooperates with SAS-6/Bld12p and facilitates assembly of the central part of the cartwheel [80].

How does SAS-5/Ana2/STIL facilitate cartwheel assembly? Biochemical and crystallographic analysis shows that the TIM domain of SAS-5 binds to a site located in the coiled-coil region of the SAS-6 dimer and suggests that SAS-5 cross-links the coiled coils of oligomerized SAS-6 and thereby facilitates SAS-6 oligomerization [81]. Given that Ana2 binds to SAS-6 in a similar manner, these proteins might assemble into a cartwheel that contains a hub and spoke with cross-links between the spokes. It is interesting to note that the position of the SAS-5-binding site on the coiled-coil tail of the SAS-6 dimer (approx. 18 nm from the hub) is close to that of the inter-spoke cross-links observed in the *Phlyctochytrium* cartwheel spoke (approx. 20 nm from the hub; figure 6*d*; see §2) [5].

This cross-linking model, however, cannot be directly applied to STIL, which binds directly to CPAP [73,74,78,79] but not to SAS-6 [75] despite the presence of a TIM domain [81]. CPAP is localized on the centriole microtubules [73,78,79], approximately 50 nm separated from the postulated cross-linking site (figure 6*d*). Based on observation that STIL rapidly shuttles between the centriole assembly site and the cytoplasm, it is proposed that STIL does not function as a cross-linker, but rather acts as a transporter that delivers CPAP and SAS-6 to a growing centriole [79]. Immunoelectron microscopy localized STIL mainly to the proximal portion of the daughter centriole [73], but its localization with respect to the cartwheel structure is not clear. More detailed localization may provide a clue to the STIL function in the assembly of the cartwheel and centriole.

A unique *in vitro* experimental system was developed about 30 years ago for analysing molecular components and assembly properties of *Tetrahymena* cartwheels. That is, spontaneous assembly of cartwheel-like structures in solution upon incubation of high-salt extracts from centrioles [28]. Compared with the SAS-6 ring formed *in vitro*, these structures look more similar to native cartwheels; they have perfect rings of the hub and tend to form stacks. This observation suggests that some factors required for the proper assembly of the cartwheel are present in mature *Tetrahymena* centrioles, and those factors are salt-extractable. Those factors are certainly worth searching for using advanced molecular biological methods currently available.

## 5. Regulation of cartwheel assembly

Assembly of cartwheels initiates new centriole formation in most organisms [82]. Therefore, this step should be tightly regulated to maintain a proper centriole number in the cell. Studies using human cells have revealed a mechanism that regulates cartwheel assembly through controlling the amount of SAS-6 in the cell. SAS-6 starts to accumulate at the end of the G1 phase and decreases in anaphase through proteasomal degradation mediated by APC/C^Cdh1^ [19]. When the SAS-6 level is artificially increased by overexpression of non-degradable SAS-6, excess procentrioles are formed on the mother centriole. Clearly, the protein level of SAS-6 is important for proper duplication of the centriole and therefore for the assembly of the cartwheel. SAS-6 is degraded by its ubiquitination also by SCF-FBXW5, a complex of an E3-ubiquitin ligase (SCF) and an F-box protein (FBXW5) that directs SCF to act on SAS-6 [83]. The activity of SCF-FBXW5 is in turn negatively regulated by the kinase activity of Plk4, the master kinase of centriole assembly. Thus, at G1/S transition, when Plk4 is activated and the cartwheel is assembled, the SAS-6 level is elevated because Plk4 inhibits ubiquitination. At the end of the M phase, when the new centriole is disengaged from the mother centriole, the SAS-6 level is lowered by degradation by the ubiquitination-proteasome system, and therefore improper centriole assembly is prevented. At the same time, the same ubiquitination-proteasome system disassembles cartwheels that have finished scaffolding roles.

In addition to the cellular amount of SAS-6, its recruitment to the mother centriole and its assembly on the site are also regulated. ZYG-1, a *C. elegans* kinase equivalent to mammalian Plk4, functions upstream of SAS-5 and SAS-6 and promotes the assembly of SAS-6 into an oligomer that could be regarded as the *C. elegans* cartwheel [71]. Mutational analyses of ZYG-1 and SAS-6 *in vivo* have revealed that ZYG-1 directly binds to SAS-6 and recruits it to the mother centriole independently of its kinase activity. The kinase activity is required for cartwheel assembly only after the SAS-6 recruitment [84]. Regarding the importance of SAS-6 phosphorylation by ZYG1 for cartwheel assembly, two groups have presented different views. One group shows that ZYG-1 promotes cartwheel assembly by phosphorylating SAS-6 at Ser 123 [85]. By contrast, the other group concludes that ZYG-1 promotes cartwheel assembly by phosphorylating a protein other than SAS-6, and that phosphorylation at any site of SAS-6 is dispensable for cartwheel assembly [84]. According to the latter group, the primary candidate for the target of ZYG-1 phosphorylation is SAS-5, which is bound to SAS-6 and required for SAS-6 oligomerization [84]. Thus, it is tempting to imagine that Plk4 regulates cartwheel assembly in mammalian cells by phosphorylating STIL.

## 6. Outlook

Our understanding of the structure and function of the cartwheel has greatly advanced in recent years, especially regarding the assembly process of its radial structure from SAS-6. However, many important questions have been left unresolved. The following are just three examples of questions. First, how is the rotational symmetry of the cartwheel restricted to ninefold? The SAS-6 ring structure assembled *in vitro* contains eight as well as nine dimers [23], and the ring in a crystal contains eight dimers [24], probably reflecting the flexibility of the SAS-6 molecule and weak association between the dimers. Additional mechanism(s) must operate to stabilize nine-membered rings. Second, how are cartwheel spokes properly arranged in radial symmetry? In contrast to the evenly spaced spokes in the cartwheel, the space between the coiled-coil tails of the SAS-6 ring assembled *in vitro* is greatly variable [23]. There may be some mechanism for proper arrangement of the spokes in the cartwheel. SAS-5/Ana2/STIL can be a primary candidate for the molecules functioning in the postulated mechanism(s) [70,72,81], but so far no solid evidence has been obtained. Third, how do the SAS-6 rings form a stack? Crystallography of the SAS-6 dimer does not predict association between the rings [23,24], suggesting that factor(s) other than SAS-6 is needed for the stacking. The molecular identity of the factors and their function must await further studies.

An obvious question regarding the centriole structure is why it has the ninefold symmetry, or, in other words, why this symmetry is so highly conserved among divergent organisms. Is it conserved because it is necessary for ciliary movement? Even the latest knowledge about the molecular mechanism for ciliary/flagellar motility cannot provide reasonable explanations for this question. To explore the meaning of ninefold symmetry in the centriole/axoneme, we need to change the microtubule number and examine the effects. Development of such an experimental system is only possible by engineering the cartwheel structure. Understanding the molecular mechanism for the cartwheel assembly will allow us to approach this fundamental question in biology.
